# Treatment with Anti-HER2 Chimeric Antigen Receptor Tumor-Infiltrating Lymphocytes (CAR-TILs) Is Safe and Associated with Antitumor Efficacy in Mice and Companion Dogs

**DOI:** 10.3390/cancers15030648

**Published:** 2023-01-20

**Authors:** Elin M. V. Forsberg, Rebecca Riise, Sara Saellström, Joakim Karlsson, Samuel Alsén, Valentina Bucher, Akseli E. Hemminki, Roger Olofsson Bagge, Lars Ny, Lisa M. Nilsson, Henrik Rönnberg, Jonas A. Nilsson

**Affiliations:** 1 Sahlgrenska Translational Melanoma Group, Sahlgrenska Center for Cancer Research, Departments of Surgery and Oncology, Institute of Clinical Sciences, University of Gothenburg, Sahlgrenska University Hospital, 40530 Gothenburg, Sweden; 2 Department of Clinical Sciences, Swedish University of Agricultural Sciences, 75007 Uppsala, Sweden; 3 Harry Perkins Institute of Medical Research, University of Western Australia, Perth, WA 6009, Australia; 4 Cancer Gene Therapy Group, Translational Immunology Research Program, Faculty of Medicine, University of Helsinki, 00290 Helsinki, Finland; 5 Department of Oncology, Comprehensive Cancer Centre, Helsinki University Hospital, 00290 Helsinki, Finland

**Keywords:** metastatic melanoma, uveal melanoma, patient-derived xenograft mouse model, adoptive T cell therapy, chimeric antigen receptor T cells, immunotherapy, canine, companion dog, comparative oncology, HER2

## Abstract

**Simple Summary:**

CAR-T cells are immune cells equipped with a claw that enable them to bind cancer cells. Usually, CAR-T cells are made using immune cells from blood. Here, we tested the hypothesis that also immune cells that reside in the tumor, so called tumor-infiltrating lymphocytes, can also be modified to carry the claw. This may mean that these cells, called CAR-TILs, will be able to attack cancer cells in two ways, using the claw or binding using its normal protein on the cell surface, the so-called T cell receptor. We show that CAR-TILs can be generated, and that they can kill melanoma cells in cell culture and in mice. Finally, to prepare for clinical trials, we also assess if CAR-TILs can be safe in a human cancer patient-like model, a companion dog suffering from cancer. Our data suggest that CAR-TILs may be a way to treat patients with melanoma but human clinical trials are needed.

**Abstract:**

Patients with metastatic melanoma have a historically poor prognosis, but recent advances in treatment options, including targeted therapy and immunotherapy, have drastically improved the outcomes for some of these patients. However, not all patients respond to available treatments, and around 50% of patients with metastatic cutaneous melanoma and almost all patients with metastases of uveal melanoma die of their disease. Thus, there is a need for novel treatment strategies for patients with melanoma that do not benefit from the available therapies. Chimeric antigen receptor-expressing T (CAR-T) cells are largely unexplored in melanoma. Traditionally, CAR-T cells have been produced by transducing blood-derived T cells with a virus expressing CAR. However, tumor-infiltrating lymphocytes (TILs) can also be engineered to express CAR, and such CAR-TILs could be dual-targeting. To this end, tumor samples and autologous TILs from metastasized human uveal and cutaneous melanoma were expanded in vitro and transduced with a lentiviral vector encoding an anti-HER2 CAR construct. When infused into patient-derived xenograft (PDX) mouse models carrying autologous tumors, CAR-TILs were able to eradicate melanoma, even in the absence of antigen presentation by HLA. To advance this concept to the clinic and assess its safety in an immune-competent and human-patient-like setting, we treated four companion dogs with autologous anti-HER2 CAR-TILs. We found that these cells were tolerable and showed signs of anti-tumor activity. Taken together, CAR-TIL therapy is a promising avenue for broadening the tumor-targeting capacity of TILs in patients with checkpoint immunotherapy-resistant melanoma.

## 1. Introduction

Patients with metastatic melanoma have a historically poor prognosis; however [1], recent advances in treatment options have drastically improved patient prognosis. Targeted therapies using inhibitors of BRAF alone [2,3] or in combination with MEK inhibitors [4,5] have shown good response rates in patients with metastatic cutaneous melanoma. However, most patients treated with these inhibitors develop drug resistance. Immunotherapies, including checkpoint inhibitors targeting PD1 and CTLA4 or LAG3, can result in more durable response rates among patients with melanoma [6,7,8,9]. Adoptive T cell transfer (ACT) with tumor-infiltrating lymphocytes (TILs) has also been used to treat metastatic melanoma in clinical trials, with response rates of approximately 50% [10,11,12]. Importantly, not all patients with metastatic malignant melanoma respond to current treatment strategies; therefore, alternative and/or combination therapies are currently being explored in preclinical experiments and trials.

Uveal melanoma (UM) is a rare form of melanoma [13] arising in the uveal tract of the eye, i.e., the iris, ciliary body, and choroid. UM is treated with brachytherapy or enucleation with very good local control (97%) [14]. However, approximately 50% of patients will later present with metastatic disease [15], mainly to the liver, but also to other sites [16]. Patients with spread UM rarely respond to systemic chemotherapy or targeted therapies, [17] and combined immune checkpoint inhibitors have not shown the same promising effect in UM [18,19] as in cutaneous melanoma [20]. Benchmark data suggest an average progression-free and overall survival of approximately 3.3 and 10.2 months, respectively [21]. Loco-regional treatment with isolated hepatic perfusion or percutaneous hepatic perfusion demonstrates high response rates and prolonged progression-free survival in randomized trials, but mature survival data are still pending [22,23]. The combined treatment with PD-1 inhibitor pembrolizumab and the HDAC inhibitor entinostat exhibited durable responses in a fraction of patients, including one with an iris melanoma with a high mutation burden and patients with tumors exhibiting a wildtype *BAP1* tumor suppressor gene [24]. ACT with TILs was tested in patients with UM in a clinical trial, with a response rate of 35% [25]. Finally, for patients with the HLA-A2 genotype, the bispecific T cell engager tebentafusp can activate anti-tumor immune responses [26] and prolong the survival of patients with UM [27], despite surprisingly low response rates [28]. Hence, although recent progress has been made, metastatic UM remains a medical challenge.

Immunotherapies aim to overcome tumor immune evasion strategies to re-activate the patient’s own immune system to attack and kill the tumor. However, some tumors downregulate the antigen presentation pathway [29,30], rendering these tumor cells insensitive to TCR-mediated recognition and cytotoxicity, thereby disarming both immune checkpoint inhibition and ACT. One way to overcome this problem is to equip T cells with a chimeric antigen receptor (CAR) designed to recognize a specific protein expressed on the surface of tumor cells irrespective of antigen presentation. CAR-T therapy has not yet been approved for use in any solid cancer; however, CD19 CAR-T therapy is used in young patients with acute lymphocytic leukemia (ALL) [31]. The reason why CAR-T therapy has not yet been successful in solid cancers is not fully understood, but includes heterogeneous expression of antigens, expression of checkpoint proteins, poor homing and tissue penetrance of CAR-T cells, an immune suppressive tumor microenvironment (TME), and CAR-T cell endurance [32].

Mouse models are useful for studying the antitumor efficacy of human CAR-T cells, but there are limitations. Patient-derived xenograft (PDX) models are immunodeficient [33], and in both PDX models and cell line-derived xenograft models, the tumor stroma and off-target tissues of CAR-T cells are of mouse origin. Therefore, these models cannot be readily used for TME or toxicity studies without additional genetic engineering. Neither xenograft nor syngeneic transplant models are spontaneous tumor models; therefore, tumor architecture can also be suboptimal. Companion dogs are an emerging and complementary model to study toxicity and anti-cancer treatment efficacy [34]. Dogs live and socialize with their human owners, share their habits and microbiota, and develop lifestyle diseases, such as cardiovascular problems, joint problems, diabetes, and cancer, with age [35]. Malignancies range from leukemia and lymphoma to solid tumors such as mammary or squamous cell carcinoma of the head and neck [36]. Melanoma is a particularly aggressive form of cancer in some breeds of dogs [37]. It is not associated with solar damage and most often develops in mucosal areas such as the mouth or under the nail bed. Similar to human mucosal melanoma, the spectrum and driver mutations of canine melanoma are different from those of human cutaneous and uveal melanoma [38,39].

We previously reported good antitumor efficacy of CAR-T cells directed against HER2 in PDX models of cutaneous and uveal melanoma [40]. We demonstrated that the elicited effect of CAR-T cells was target-specific, as CRISPR/Cas9-mediated disruption of HER2 abolished the sensitivity of melanoma cells to anti-HER2 CAR-T cells. Importantly, CAR-T cells were also able to eradicate tumors that were refractory to ACT. Current CAR-T therapies use CAR-transduced T-cells from blood as drug substances. This T cell pool largely consists of naïve and memory T cells carrying a TCR with irrelevant affinity. Naïve T cells generally do not express molecules that facilitate homing to inflamed peripheral tissues. TILs, on the other hand, can home to tumors; therefore [41,42], they could potentially serve as good starting materials for the generation of CAR-T cells. The aim of this study was to assess whether CAR expression in TILs can boost tumor cell death. We also assessed whether the CAR-TILs were safe and tolerable in mice and companion dogs.

## 2. Materials and Methods

### 2.1. Human Patient Samples

Tumor samples were obtained from patients treated at the Department of Surgery, Sahlgrenska University Hospital, Gothenburg, Sweden, following informed consent (Regional Human Ethics Board of Västra Götaland, Sweden approval #288-12 and #44-18). Tumor cells were extracted from tumor samples and used for patient-derived xenograft establishment, as previously described [43]. Young TILs (y-TILs) were extracted from the same tumor samples, cultured, and expanded, as previously described [42].

### 2.2. Cell Experiments

Patient-derived melanoma cell lines were maintained in RPMI with 10% fetal bovine serum and were either described previously [UM22, MM2, MM3, MM4 [42,44]] or generated by culturing melanoma samples in RPMI with 10% FCS (MM5, MM6). 92-1 Uveal melanoma cells were a kind gift from the European Collection of Authenticated Cell Cultures (ECACC, UK, or available from Sigma-Aldrich), and the HER2 knockout line was established previously [40]. The canine tumor cell lines D17.os and CF41.mg were purchased from ATCC (Manassas, VA, USA).

For CRISPR/Cas9 inactivation of *B2M*, the Cas9:crRNA:tracrRNA ribonucleoprotein (RNP) complex was assembled according to the manufacturer’s recommendations (IDT DNA) and transfected into the cells using Lipofectamine RNAiMAX reagent (Invitrogen, Thermo Fisher, Carlsbad, CA, USA). Negative cells were sorted based on the absence of B2M-PE antibody staining (clone 2M2, BioLegend, San Diego, CA, USA) using magnetic separation with PE-beads (Miltenyi, Bergisch, Germany), confirmed negative by staining with the same antibody, and analyzed using an Accuri C6 flow cytometer (BD, Franklin Lakes, NJ, USA) equipped with the BD Accuri C6 softwaren (v1.0).

### 2.3. Generation of TILs and CAR-TILs

Young TILs (yTILs) were made by cutting 2–3 mm^3^ pieces from tumors and placing these in a 24-well plate containing RPMI medium supplemented with 10% human AB serum (Sigma-Aldrich, St. Louis, MO, USA), 6000 IU/mL human recombinant IL2 (Peprotech, East Windsor, NJ, USA), 1 mM sodium pyruvate (Gibco, Thermo Fisher, Carlsbad, CA, USA), and 50 µM 2-Mercaptoethanol (Gibco). After 10–14 days, the yTILs were either expanded or cryopreserved for future use. For the rapid expansion (REP) of TILs, yTILs (1 × 10^5^) were mixed with irradiated (40 Gy) feeder cells (20 × 10^6^), CD3 antibody (clone OKT3, 30 ng/mL, Miltenyi), and a medium containing 50% RPMI and 50% AIM-V (Invitrogen) supplemented with 10% human AB serum (Sigma-Aldrich) and 6000 IU/mL IL-2 (Peprotech).

A HER2 CAR-expressing lentiviral vector that was previously shown to be specific for HER2 was purchased from ProMab (Richmond, CA, USA) [40]. The second-generation CAR expressed from this vector contains a CD8 leader, a Herceptin-like scFV binding domain, a CD8 transmembrane region and the intracellular signaling domains from CD3ζ and CD28. CAR-TILs were produced by transducing TILs on days 1 and 2 of the REP with the HER2 CAR lentiviral vector in the presence of Vectofusin-1 (Miltenyi). After five days of culture at 37 °C in 5% CO_2_, half of the medium was replenished. From day six onwards, the flasks were inspected daily and split when necessary to maintain cell densities of approximately 1–2 × 10^6^/mL. After 14 days in culture, the cells were harvested, resuspended in PBS with 300 IU/mL of IL-2, and intravenously transplanted into mice (20 × 10^6^ cells per mouse in 100 µL).

Alternatively, REP-TILs were transfected with anti-HER2 CAR mRNA via electroporation using a 4D nucleofector (Lonza, Basel, Switzerland). This mRNA was generated by PCR amplification of the coding sequence of anti-HER2-CAR [40] using primers that carried a T7 RNA polymerase recognition sequence at the 5′-end. The resulting PCR product was used in an in vitro transcription reaction with the T7 mScript Standard mRNA Production System (Cellscript, Madison, WI, USA). The TILs were resuspended in P3 primary cell solution with supplement (Lonza), and mRNA was added before pulsing using the DN100 program.

### 2.4. CAR Detection

For qPCR detection, genomic DNA was prepared from TILs and CAR-TILs 10–14 days after the start of REP by lysing in Direct PCR Lysis Reagent (Nordic BioSite, Stockholm, Sweden) and proteinase K. Quantitative PCR was performed in triplicate using a qPCR SyGreen mix (Techtum Lab AB, Stockholm, Sweden), and the PCR reaction was performed with a CFX cycler (Bio-Rad, Hercules, CA, USA). Data analysis comparing ΔCT values normalized to a reference gene (β-actin) was performed to determine the CAR copies/cell.

For analysis of the HER2 protein binding capacity of CAR-TILs, 100,000 cells were incubated with 1 µg biotinylated HER2 protein (Abcam, Cambridge, UK) for 30 min at 4 °C, followed by incubation with an allophycocyanin-conjugated streptavidin antibody (Jackson Immuno Research, West Grove, PA, US) for 25 min at 4 °C, and flow cytometry analysis was performed.

### 2.5. Cytotoxicity Experiments

Melanoma cells were infected with a lentivirus made using pHAGE-PGK-GFP-IRES-LUC-W (Addgene # 46793) containing coding sequences for green-fluorescent protein and firefly luciferase. To assess the cytotoxicity of T cells, patient-derived melanoma cell lines expressing luciferase were plated at 20,000 cells/well in black 96-well plates (Corning, Corning NY, USA) and cultured in the presence or absence of different ratios of T cells per well. After 48 h, the medium was aspirated for IFN-γ secretion analysis using an ELISA kit (Diaclone, Besancon Cedex, France), and the viability of the cancer cells was assessed by measuring luminescence with a GloMax Discover plate reader (Promega, Madison, WI, USA) or an IVIS Lumina III XR (Perkin-Elmer, Waltham, MA, USA) after adding luciferin (150 µg/mL) to the cells. For degranulation analysis, TILs and CAR-TILs were co-cultured with cancer cells for 4–6 h in RPMI supplemented with human AB serum (Sigma-Aldrich) and CD107a antibody (clone H4A3, BD), followed by washing in PBS and the detection of bound CD107a antibody by flow cytometry (BD Accuri C6 Plus). For the IFN-γ secretion assay, the degranulation assay was followed by additional staining procedures according to the manufacturer’s instructions (Miltenyi Biotech), and bound IFN-γ was detected using flow cytometry. For the detection of cleaved caspase 3 (CC3), cancer cells were co-cultured with TILs and CAR-TILs for 24 h, followed by fixation and permeabilization using Cytofix/Cytoperm solution for 25 min (554714, BD), washed twice with supplemented Wash/Perm buffer (554714, BD), and stained with a CC3 antibody (clone C92-605, BD) at 1:10 dilution in Wash/Perm buffer. Finally, the cells were washed and analyzed using flow cytometry.

### 2.6. Mouse Experiments

All mouse experiments were performed in accordance with EU Directive 2010/63 (Regional Animal Ethics Committee of Gothenburg #2014-36, #2016-100, and #2018-1183). Non-obese diabetic-severe combined immune-deficient interleukin-2 chain receptor γ chain knockout mice (NOG mice, Taconic, Ry, Denmark) and human IL-2 transgenic NOG (*hIL2*-NOG) mice (Taconic) were used for the engraftment of tumor samples. Tumor size was monitored by caliper measurements, alternatively bioluminescent signals, using the IVIS imaging system (Perkin-Elmer). When tumor growth was confirmed by two consecutive measurements, *hIL2*-NOG mice were treated with autologous human tumor-infiltrating lymphocytes (TILs; 20 million cells per mouse) by intravenous injection into the tail vein. NOG mice served as untreated controls.

### 2.7. Canine CAR-TIL Expansion

For canine yTIL expansion, 2–3 mm^3^ tumor pieces were cut and placed in a 24-well plate containing RPMI medium supplemented with 10% human AB serum (Sigma-Aldrich), 6000 IU/mL human recombinant IL2 (Peprotech), 1 mM sodium pyruvate (Gibco), and 50 µM 2-Mercaptoethanol (Gibco). After seven days, the yTILs were harvested, washed in PBS, and resuspended in PBS supplemented with 10% fetal bovine serum. Cells were stained with a CD5-PE antibody (clone YKIX322.3, eBioscience) diluted 1:50 for 20 min at 4 °C, washed in PBS, and stained with PE microbeads (Miltenyi) according to the manufacturer’s protocol. Finally, cells were washed and CD5 positive cells were sorted using MACS separation with LD columns (Miltenyi) using a QuadroMACS Separator (Miltenyi) according to the manufacturer’s instructions. CD5 positivity was confirmed by analyzing PE expression using flow cytometry (BD Accuri C6 Plus). CD5 positive yTILs were either cryopreserved for later expansion or directly expanded using a rapid-expansion protocol (REP). For REP of canine TILs, CD5 positive yTILs (1 × 10^5^) were mixed with 20 × 10^6^ irradiated (40 Gy) feeder cells from healthy dog blood donors (three different donors) and human CD3 antibody (clone OKT3, 30 ng/mL, Miltenyi) and expanded for 14 days in RPMI supplemented with 10% human AB serum (Sigma-Aldrich), 6000 IU/mL human IL2 (Peprotech), 1 mM sodium pyruvate (Gibco), and 50 µM 2-Mercaptoethanol (Gibco). For CAR-TIL production, cells were electroporated with anti-HER2 CAR mRNA after expansion and used to treat the patient the next day. Alternatively, anti-HER2 CAR lentivirus was added after 1, 2, and 12 d of culture.

### 2.8. First-in-Dog (FIDO) Trial

Four client-owned dogs with HER-2 positive aggressive tumors were recruited at the University Animal Hospital (Uppsala, Sweden) between November 2019 and April 2021. All the dogs had recurrent spontaneous cancers with metastases. The CAR-TIL FIDO study and sample collection were approved by the Swedish Animal Ethical Committee and Swedish Animal Welfare Agency (#2019-2435). Written consent was obtained from dog owners.

The study design was as follows: at the initial visit, blood samples were collected and the hematology and biochemistry were analyzed. The dogs were screened with a whole-body CT scan (dogs 1 and 4) or thoracic X-rays and ultrasound of the local lymph nodes and abdomen (dogs 2 and 3). As soon as possible, surgical extirpation of the primary tumor was performed in three dogs (dogs 1–3) and of the metastasized local lymph nodes in one dog (dog 4). The tumors were sent to a pathology laboratory for diagnostic purposes. Parts of the tumors were sent in sterile PBS containing Primocin (InVivoGen, San Diego, CA, USA) to the laboratory in Gothenburg for expansion of autologous TILs. The TILs were modified, grown, and harvested as described below.

Before the first dose of CAR-TILs, each dog was treated for 10–14 days with oral Toceranib phosphate (Hospital pharmacy, brand name Palladia^TM^) 2.3–2.5 mg/kg *qd* to suppress regulatory T-cells. Treatment was stopped five days before CAR-TIL transfusion. The dogs were sedated with subcutaneous injections of medetomidine:butorphanol (0.01 mg/kg:0.1 mg/kg). Transfusion of CAR-TILs was performed via a peripheral venous catheter for 30 min using a protocol that included monitoring vital signs (body temperature, breathing, mucous membrane heart rate, blood pressure, and pulses). After the transfusion, the sedated dogs were reverted with an intramuscular injection of atipamezol (0.05 mg/kg). Dogs stayed at the clinic for at least one hour after transfusion for supervision. Three dogs were treated with amoxicillin 9.5–11.5 mg/kg bidaily (BID) for 10 days and 1 (dog 2) was treated with clindamycin 12 mg/kg BID for 10 days, after each CAR-TIL treatment. Human IL-2 (a kind gift from the NCI) was administered by subcutaneous injections.

Blood samples were collected at visits for treatment and revisits. A complete blood count, standard clinical chemistry profile, and immunoglobulin gel electrophoresis were performed. Response to therapy was categorized in accordance with veterinary-adjusted RECIST criteria [45] as CR (complete regression of measurable soft tissue disease), PR (partial response of at least 30% reduction in the sum of diameters of target lesions, taking as reference the baseline sum), PD (progression of the disease by either the appearance of one or more new lesions or at least a 20% increase in the sum of diameters of target lesions, taking as reference the smallest sum on study), and SD (less than 30% reduction or 20% increase in the sum of diameters of target lesions, taking as reference the smallest sum of diameters while on the study). The best overall response was defined as the best response recorded from the start of treatment to disease progression or recurrence. Macroscopic tumor lesions were measured using a caliper and documented using photographs. In addition, dogs were followed up with diagnostic imaging (computed tomography, radiography, and/or ultrasound), using the most suitable modality for each case. Adverse events were graded according to VCOG-CTCAE version 2 [46].

### 2.9. Protein Analysis

For immunohistochemistry, dog tumor tissues were fixed in 4% formalin, dehydrated, and embedded in paraffin. Sections (4 μm were mounted onto positively charged glass slides and dried at 60 °C for 1 h. The slides were rehydrated, and antigen retrieval was performed by heat-induced epitope retrieval (HIER) in Dako PT Link with a high pH buffer (Dako, Glostrup, Denmark). The staining was performed with an autostainer (Autostainer Link 48, Dako) using the following protocol: Endogenous Enzyme Block for 5 min (FLEX Peroxidase Block, Dako), primary antibody (HER2 A0485 and Melan-A IR633; both from Dako) staining for 60 min at room temperature, secondary reagent staining (FLEX + Rabbit LINKER, K8009, Dako) for 15 min, FLEX HRP for 20 min, diaminobenzidine (DAB) chromogen development for 10 min, and counterstaining with hematoxylin for 12 min. The slides were dehydrated and mounted using a Pertex.

### 2.10. Statistical Analyses of Experimental Data

**Figure 1:**
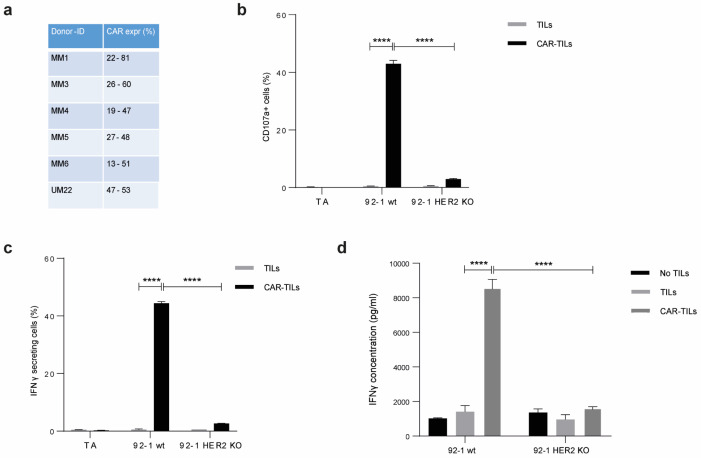
TILs can be activated by equipping them with a CAR construct. mRNA encoding anti-HER2 CAR was electroporated into TILs from five cutaneous melanomas (MM1, MM3, MM4, MM5, and MM6) and one uveal melanoma (UM22) to produce CAR-TILs. CAR expression was detected by HER2-biotin binding at 5–16 h post-transfection (**a**). MM1 TILs and CAR-TILs were co-cultured with the parental or HER2 KO 92-1 uveal melanoma cell line for 4–6 h, followed by flow cytometry to measure degranulation (CD107a expression) (**b**) or IFN-γ-secreting cells (**c**). TILs and CAR-TILs not co-cultured with 92-1 cells (TILs alone; TA) were used as controls in (**b**,**c**). Alternatively, cells were co-cultured for 48 h, and the supernatant was collected for analysis of secreted IFN-γ by ELISA (**d**). 92-1 cells not co-cultured with TILs (no TILs) were used as a control. Data are presented as mean ± standard deviation (SD) of duplicates. The experiments were performed twice, and representative results are shown. *p* values are represented as **** *p* < 0.0001.

**Figure 2:**
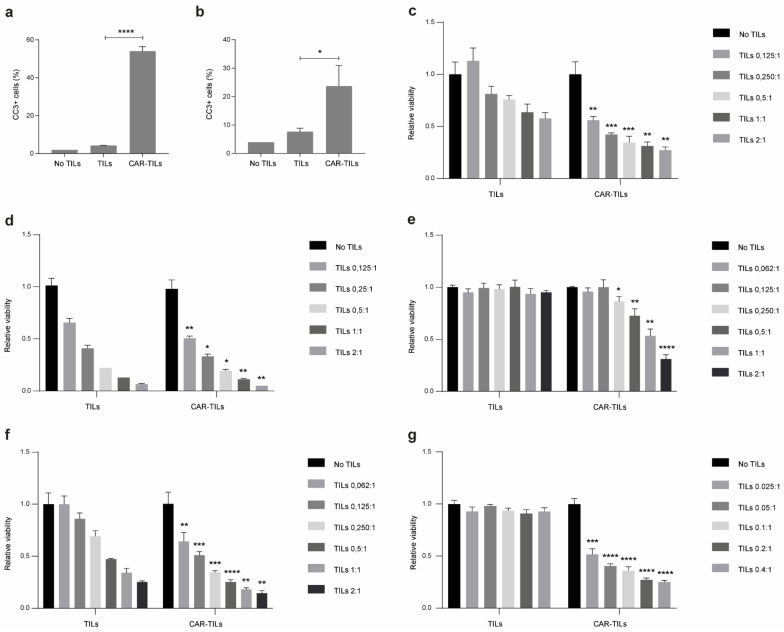
CAR-TILs kill tumor cells more efficiently than TILs. (**a**,**b**) TILs and CAR-TILs from MM1 were co-cultured with the parental or HER2 KO 92-1 uveal melanoma cell line for 24 h, followed by cleaved caspase 3 (CC3) detection in the tumor cells using flow cytometry (**a**). Additionally, TILs and CAR-TILs from MM5 were co-cultured with autologous cancer cells for 24 h, followed by cleaved CC3 detection using flow cytometry (**b**). Cancer cells not treated with TILs were used as a control (no TILs). (**c**–**g**) Viability of autologous cancer cells was measured by luciferase signal detected after 48 h co-culture with increasing doses of TILs and CAR-TILs from UM22 (**c**), MM3 (**d**), MM4 (**e**), MM5 (**f**), and MM6 (**g**) in the indicated ratios (TILs:cancer cells). Data are presented as mean with SD of triplicates. Asterisks represent *p*-values of difference between similar doses of TILs and CAR-TILs. The experiments were performed twice, and representative data from one experiment is shown. P values are represented as * *p* < 0.05, ** *p* < 0.01, *** *p* < 0.001, and **** *p* < 0.0001.

**Figure 3:**
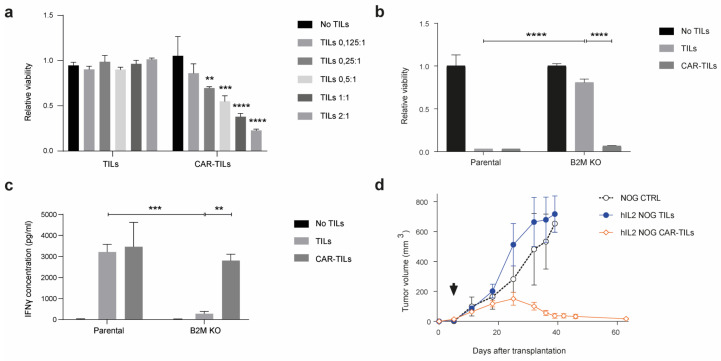
Anti-HER2 CAR-TILs eradicate autologous tumor cells refractory to TCR-mediated cytotoxicity. Parental MM3 melanoma and MM3 cells with CRISPR-Cas9 disruption of β-2-Microglubulin (B2M KO, Supplementary Figure S5) were cultured with autologous TILs or CAR-TILS. (**a**,**b**) Viability was measured by luciferase activity in melanoma cells after 48 h of co-culture with TILs or mRNA generated (**a**) or virus generated (**b**) CAR-TILs. (**c**) IFNγ was measured in the culture supernatant from the same experiment as in (**b**). (**d**) Mice bearing B2M CRISPR knockout MM3 melanoma cells were treated with PBS (*n* = 3), 20 × 10^6^ TILs (*n* = 3), or 20 × 10^6^ CAR-TILs (*n* = 3). Arrow indicates treatment start. Data are presented as mean ± standard error of the mean. *p* values are represented as ** *p* < 0.01, *** *p* < 0.001, and **** *p* < 0.0001.

Statistical analysis was performed using GraphPad software, ordinary one-way ANOVA, multiple comparisons with Tukey’s correction (Figure 1 and Figure 3b,c), and alternatively unpaired *t* tests (Figure 2 and Figure 3a). P values are represented as * *p* < 0.05, ** *p* < 0.01, *** *p* < 0.001, and **** *p* < 0.0001. All mouse experiments contained 3–4 mice per group.

### 2.11. Single Cell Gene Expression Analysis

Single-cell RNA-seq from two recent studies [24,44] was used to compare the gene expression profiles of CD8^+^ and CD4^+^ T cells found in tumors (TILs) and blood from patients with uveal melanoma. Alignment and estimation of gene expression levels were performed using Cell Ranger (v. 3.0.2, 10× Genomics). The specific commands used were *cellranger count* (with the 10× Genomics version of the GRCh38 reference transcriptome; v. 3.0.0) and *cellranger vdj* (with the 10× Genomics GRCh38 VDJ reference dataset, v. 2.0.0). After identifying cell types, the remaining analyses were performed using the *Seurat* R package (v. 4.0.3) [47], and data were imported and normalized using the *NormalizeData* function with default settings. Cells predicted to be duplicates were excluded from statistical tests using R package *DoubletFinder* (v. 2.0.3, parameters: PCs = 1:15, pN = 0.25). Cells with more than one TCR alpha or beta chain were also excluded; however, not all cells were duplicates. An approach described by Karlsson et al. [44] was used to classify the cell types in both datasets. Differential expression was assessed using the Seurat FindMarkers function (test.use = “LR,” logfc.threshold = −Inf) between the same cell type in the two datasets. Note that the batch effects cannot be excluded from the analysis. FindMarkers were run at three different times with or without accounting for the cell cycle (derived from the Seurat function CellCycleScoring) and sex, and only genes commonly identified as differentially expressed in all three analyses were retained (Supplementary Table S3).

### 2.12. RNA-Sequencing

RNA was prepared from companion dog tumors using the RNA/DNA kit from Qiagen. RNA was sequenced at the Clinical Genetics Center at Sahlgrenska University Hospital using a Novaseq sequencer. Reads were aligned to the CanFam3.1 genome (http://ngi-igenomes.s3.amazonaws.com/igenomes/Canis_familiaris/Ensembl/CanFam3.1/Sequence/WholeGenomeFasta/genome.fa, accessed on 5 February, 2021) with STAR (v. 2.7.10a) [48], with a matching reference genome annotation supplied from AWS iGenomes (http://ngi-igenomes.s3.amazonaws.com/igenomes/Canis_familiaris/Ensembl/CanFam3.1/Annotation/Genes/genes.gtf, accessed on 5 February 2021), using the parameters “twopassMode Basic” and “setting—sjdbOverhang” equal to read length 1. Gene expression levels were quantified from name-sorted and non-duplicate marked alignment files using htseq-count (v. 1.99.2) [49], with the parameters “-s reverse -m intersection-strict”.

### 2.13. Transcriptomic Classification

TCGA data, downloaded and processed as described previously [50], were used for the classification of canine tumors relative to human cancer types. Pairwise Spearman correlation coefficients were calculated between our sample and each TCGA sample for all coding genes. Classification was performed using a k-nearest neighbor approach based on these correlation coefficients, using *k* = 6, as previously found to be optimal based on leave-one-out cross-validation in TCGA cohort [50].

## 3. Results

### 3.1. TILs Differentially Express Chemokine Receptors and Selectins

CAR-T cells were approved for clinical use against some B-cell malignancies; however, no CAR-T cell therapy has been approved for solid tumors. To investigate the difference in the expression of cell surface molecules involved in T cell trafficking and homing, we performed single-cell sequencing of blood-derived T cells and young TILs that had grown out uveal melanoma metastases in the presence of the T cell growth factor interleukin-2 (IL-2) [44]. Focusing on differentially expressed genes in the category of ‘chemokine receptors’ or ‘adhesion molecules’ we find that genes like ITGA4 (CD49D), ITGB2 (CD18), ITGAL (CD11a), ITGAE (CD103), PECAM1 (CD31), and CXCR3 are all more highly expressed on TILs compared to on blood-derived T cells (Supplementary Figure S1a). Since TILs are cultured, we validated the finding at the protein level using flow cytometry. Although it confirmed the differential expressions, it also demonstrated that at least some of this differential expression could be caused by the IL-2 used in the culture medium of TILs (Supplementary Figure S1b,c).

### 3.2. CAR-TILs Can Kill Melanoma Cells In Vitro

CAR-T cell manufacturing is performed by modifying blood-derived T cells from leukapheresis with a chimeric antigen receptor (CAR) that binds to a surface antigen on the tumor cell. To investigate whether anti-HER2 CAR-expressing TILs, hereafter called CAR-TILs, are capable of recognizing melanoma cells, we used TILs from human patients with melanoma and transfected these cells with an mRNA encoding a HER2 CAR consisting of a single-chain variable fragment (scFv) to detect HER2 fused with the signaling domains of CD3 epsilon and the CD28 co-stimulatory molecule [40]. We consistently observed HER2 CAR expression in these cells which peaked at 5–16 h but lasted for at least 24 h, as assessed by recombinant HER2 binding to CAR-TILs (Figure 1a and Supplementary Figure S2). CAR was functional and specific because the CAR-TILs (but not mock-transfected TILs) from patient MM1 degranulated, released, and caused accumulation of interferon gamma (IFN-γ) in the medium when co-cultured with the HER2 positive uveal melanoma cell line 92-1 (Figure 1b–d). This was dependent on HER2 expression since CRISPR-generated 92-1 HER2 knockout cells [40] were not able to activate CAR-TILs. This demonstrates that anti-HER2 CAR-TILs can react with HER2 on the surface of melanoma cells.

Next, we co-cultured MM1 or CAR-TILs with 92-1 cells and stained them with an antibody directed against cleaved caspase-3. 92-1 cells cultured with CAR-TILs, but not TILs, were positive for intracellular cleaved caspase-3, suggesting that the cells underwent apoptosis (Figure 2a). We also generated patient-derived cell lines, TILs, and CAR-TILs from MM5 cells. CAR expression in autologous MM5 TILs enhanced their ability to kill melanoma cells (Figure 2b). Four out of five patient-derived cell lines were more sensitive to increasing amounts of CAR-TILs than to TILs in the co-culture experiments (Figure 2c–g). This correlated with the greater degranulation of CAR-TILs than mock-transfected TILs (Supplementary Figure S3).

### 3.3. CAR-TILs Can Kill Melanoma Cells In Vivo

The mRNA transfection of CAR into TILs allowed for a fast way to assess the efficacy of CAR in TILs, but the expression was not durable. TILs kill via the binding of the T-cell receptor (TCR) to the MHC class I complex, which is loaded with peptides. The MHC complex consists of one alpha chain (encoded by HLA A/B/C) and one beta-2-microglobulin (B2M) chain (encoded by B2M). To challenge the TILs, we deleted B2M by CRISPR/Cas9 (B2M KO; Supplementary Figure S4) in a cell line from patient MM3, rendering the tumor cells resistant to the cytotoxic activity of autologous TILs but not to anti-HER2 CAR mRNA-transfected CAR-TILs (Figure 3a).

To study the effect of CAR-TILs in vivo we used a lentivirus expressing the same anti-HER2 CAR mRNA [40]. CAR expression was much lower in lentivirus-transduced cells than in those transfected with CAR mRNA and only 1–10% of TILs were susceptible to lentivirus transduction (Supplementary Figure S5). Nevertheless, this modification of MM3 TILs enabled the cytotoxicity of both parental MM3 cells and B2M KO tumor cells in vitro (Figure 3b), which correlated with IFN-γ secretion into the medium.

We previously showed that TILs and blood-derived HER2 CAR-T cells can eradicate PDX tumor models in the human IL-2 transgenic NOG/NSG mouse strain (hIL2-NOG). We transplanted B2M KO MM3 cells into hIL2-NOG cells and treated them with TILs or anti-HER2 CAR-TILs. As expected from the in vitro experiments (Figure 3a–c), TILs were not able to eradicate B2M deficient melanoma in the PDXv2 model, but CAR-TILs could (Figure 3d). In PDX models from UM1, MM2, and MM7, the added value of lentiviral CAR expression in TILs was only visible in MM2, since unmodified TILs could eradicate tumors in UM1 and MM7 PDX models (Supplementary Figure S6).

### 3.4. First-in-Dog (FIDO) Trial Suggests CAR-TIL Therapy Is Safe and May Have Anti-Tumoral Activity in Tumor-Bearing Companion Dogs

**Figure 4:**
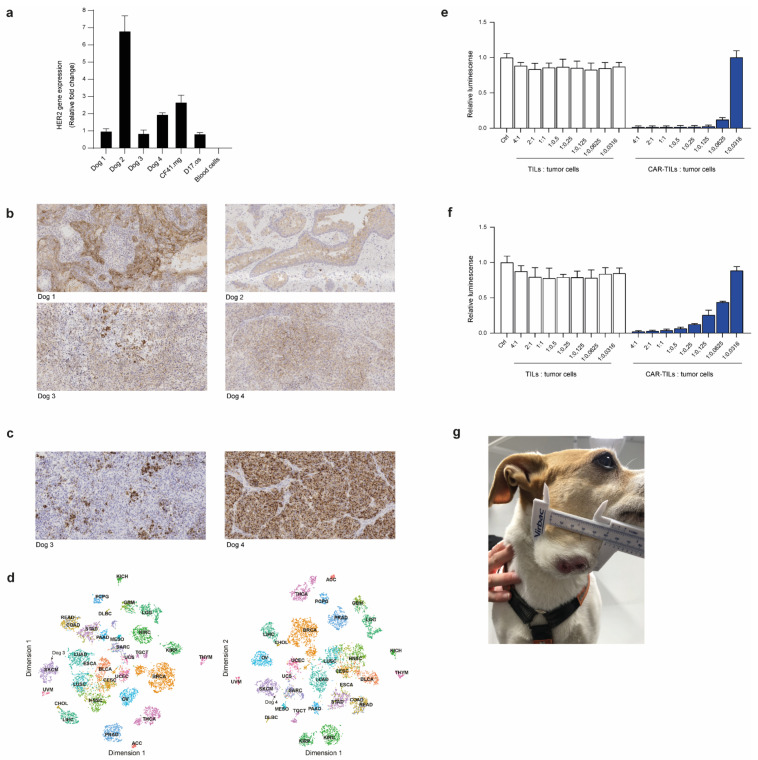
Dog tumor cells express HER2 and can be killed by anti-HER2 CAR-TILs. HER2 expression detected by qPCR (**a**) and immunohistochemistry (**b**) in tumors from four companion dogs (dogs 1-4). Canine cell lines D17.os and CF41.mg and canine PBMC were used as controls in a. (**c**) Expression of human melanoma marker Melan-a in tumors from dogs 3 and 4 with melanoma. (**d**) Classification of canine tumor biopsies from dogs 3 and 4 based on gene expression relative to TCGA cohort of >10,000 human tumor samples from 32 cancer types. The similarities between canine melanoma and samples in TCGA were visualized using tSNE dimensionality reduction. SKCM: melanoma cases. (**e**,**f**) TILs and mRNA electroporated CAR-TILs from dog 3 were co-cultured with the canine cell lines CF41.mg (**e**) and D17.os (**f**) for 24 h. The experiment was performed twice and representative data (*n* = 3, data ± SD) from one experiment are shown. (**g**) Photograph of Dog 3 with metastatic oral malignant melanoma enrolled in the FIDO trial evaluating the safety of CAR-TIL treatment in companion dogs.

To assess the safety of anti-HER2 CAR-TILs in an immune-competent and spontaneous tumor model before the first-time-in-man trial (FTIM), we conducted a first-in-dog (FIDO) trial that recruited four companion dogs with high-grade cancer (two with squamous carcinoma and two with melanoma). In parallel with the recruitment and treatment of dogs, we also performed several analyses of the tumors and TILs. First, we assessed the expression of HER2 in the tumors of all dogs compared to blood leukocytes, canine D17.os osteosarcoma and CF41.mg mammary cell lines. The Dog 2 tumor had the highest HER2 mRNA expression, followed by the mammary cell line and the second melanoma dog (Dog 4). The tumors of dogs 1 and 3 had a similar expression as the osteosarcoma line, whereas blood leukocytes were practically negative for HER2 expression (Figure 4a). Immunohistochemistry demonstrated HER2 expression in all dog tumors (Figure 4b).

We recruited two dogs with melanoma, Dog 3, which had a patchy expression, and Dog 4, which had a uniform expression of the melanoma marker Melan-a (Figure 4c). To compare the transcriptomes of canine and human cancers, we used an in-house bioinformatics pipeline developed to diagnose cancer of unknown primary origin [50]. By comparing the gene expression of around 10,000 human tumors in The Cancer Genome Atlas (TCGA) with those of the biopsies from dogs 3 and 4 using k-nearest neighbor analysis, we could analyze the similarities between the canine melanomas to other cancers. Of the 10 tumors whose transcriptome correlated to that of dog 3′s tumor (Spearman correlation 0.8), 8 were melanomas. The other two tumors were those of breast cancer and bladder cancer. In total, 5 of the 10 most similar tumors to Dog 4′s tumor were melanomas, the remaining being 2 sarcomas, 1 breast cancer, 1 bladder cancer, and 1 esophageal tumor. The similarity to human melanoma (SKCM) with all tumors in TCGA are also visible in tSNE plots (Figure 4d).

To evaluate whether our anti-HER2 CAR binder would bind to canine HER2, we transfected canine TILs with the mRNA encoding anti-HER2 CAR. We co-cultured the TILs or anti-HER2 CAR-TILs with the canine D17.os and CF41.mg cell lines labeled with firefly luciferase. Since luciferase requires ATP to glow, we were able to assess the viability of tumor cells exclusively by luciferase measurements when co-cultured with increasing amounts of TILs or CAR-TILs. None of the cell lines were sensitive to allogenic TILs, but both were killed by CAR-TILs in a dose-dependent manner (Figure 4e,f). The canine mammary carcinoma cell line was more sensitive than the osteosarcoma cell line, suggesting that the level of HER2 expression (Figure 4a) affects sensitivity to CAR-TILs.

The FIDO trial design was a dose-escalation study giving 0.1–10 million CAR-TILs/kg dog, without or with injections of human IL-2. All dogs received one dose of the lowest dose of CAR-TILs, and after safety monitoring, one or two additional doses with or without IL-2 were administered. The first two dogs had squamous cell carcinoma of the tongue and tonsils. The dogs initially tolerated the treatment well; however, the first dog developed pyometra, a common disease in female dogs of this breed. None of the other two female dogs in the study developed this disease, suggesting that it was not apparently related to treatment. The first dog experienced a slight reduction in tumor size, but this was not a durable response (Supplementary Table S1). Since the percentage of anti-HER2 CAR positive cells was very low in the CAR-TILs of the first dog, and the main purpose of the trial was to evaluate the safety of the anti-HER2 CAR binder, we used anti-HER2 CAR mRNA transfection for the next dog. We achieved approximately 30% of CAR-positive cells. The tumor size was not accurately measurable from the back of the tongue, but there was no apparent softening or decrease in size with treatment, and the dog was euthanized upon tumor progression because of deteriorating quality of life (Supplementary Table S2).

Dogs 3 and 4 suffer from melanoma, a debilitating disease in dogs because it often affects eating: it rapidly develops systemic metastases and, if untreated, is lethal. We were able to generate more TILs from dogs with melanoma and were therefore able to inject both mRNA-transfected and virus-transduced CAR-TILs, as well as treatment with IL-2. Initially, the tumor grew rapidly on Dog 3 (Figure 4g), but a transient decrease in tumor size (PR) was observed after the third CAR-TIL injection and first IL-2 injections. The dog experienced gastrointestinal side effects, such as loss of appetite, vomiting, and diarrhea (Table 1), which were resolved and did not appear after subsequent reduction in the dose of reminders of IL-2 injections. After the initial decrease in tumor size, the tumor progressed, and the dog was euthanized because of tumor progression.

The fourth dog underwent surgery for subungual melanoma with metastasis to the prescapular lymph node, and no detectable tumor was detected after surgery. Nevertheless, since local recurrence or distant metastases invariably develop within 3–6 months in these cases, we treated the dog with both the lowest and therapeutic dose of CAR-TILs in an adjuvant setting. No acute side effects were observed with CAR-TIL treatment; however, after IL-2 treatment, gastrointestinal side effects and eosinophilia resolved with treatment discontinuation and re-emerged when reinitiating treatment (Table 2). IL-2 treatment ceased, and one year after surgery, the dog was still tumor-free.

Monitoring of safety in all four dogs throughout the treatment period did not reveal any apparent effects on blood pressure or cardiac, pulmonary, or endocrine functions. Liver and electrolyte levels were normal, but CRP was elevated in response to tumor progression, IL-2 administration, or infection (Supplementary Tables S1 and S2, and Table 1 and Table 2).

## 4. Discussion

Immunotherapies with checkpoint blockade have revolutionized the treatment of metastatic melanoma, with durable and high response rates in patients who previously had a poor prognosis. However, some tumors pose a challenge for immunotherapies because of their low antigen load or different immune evasion strategies. A great challenge in the field is how to render these tumors immunogenic.

We posed the question of whether it would be possible to enhance the effect of TIL therapy by genetic engineering with CAR. Indeed, we showed that this therapeutic approach resulted in the activation of T cells that could not kill allogenic or immune-evaded autologous tumors. We observed complete tumor regression in PDX mice from tumors that lacked B2M and were treated with CAR-TILs (but not TILs). These responses were superior to those observed in a study published while writing this manuscript [51]. This can be explained by the use of different immunocompromised mouse strains. Our data confirm the importance of IL-2 supplementation for TIL and CAR-T activity in vivo as shown in hIL2-NOG mice [40,42]. On the other hand, Mills et al. [51] reported better transduction efficiencies using CAR-expressing retroviruses, although they selected cells to ensure high expression levels. This information is valuable for advancing the concept of CAR-TILs in the clinic.

CAR-TIL treatment could be used in patients with cancers that are normally not recognized by the immune system, for instance, due to downregulation of MHC or the antigen presentation machinery, lack of suitable neoantigens, or expression of any inhibitory receptors (including but not limited to PD1 and CTLA-4). One PDX model, which is a poor responder to TIL therapy (MM2), was rendered more responsive to T-cell cytotoxicity after equipping the TILs with a CAR construct. However, tumors that respond to ACT treatment with autologous TILs (e.g., UM1 and MM7 in this study) may not be eradicated more efficiently by CAR-TIL treatment. Hence, a potential target population for a trial could consist of patients with immune checkpoint inhibitor-resistant metastatic cutaneous, mucosal, or uveal melanoma.

A challenge for immunotherapy research is to utilize good preclinical models to study human tumors and to generate preclinical efficacy and safety data that can warrant the start of clinical trials. Most preclinical advances have depended on in vitro studies of human tumors and immune cells, mouse tumor models, and mouse immune cells. In vitro systems do not always recapitulate the in vivo setting in a satisfactory manner, highlighting the importance of using in vivo models to develop novel treatment strategies for cancer patients. Mouse models used to study mouse tumors and immune cells accurately recapitulate the complex truth [52], but with limitations attributed to differences between mouse and human biology. The hIL2-NOG mouse model used here [42] has the advantage of recapitulating the heterogeneous nature of TIL therapy in cancer patients, which is not possible in NOG mice. Unfortunately, for the rare form of melanoma arising in the eye, uveal melanoma, there is a lack of models, and those that we have developed [40,44] most often grow slower in NSG/NOG mice than in cutaneous melanoma.

Companion dogs are emerging models for human diseases because their etiologies are very similar [34,36]. In this study, we utilized the fact that solid tumors in companion dogs express the dog variant of HER2, which was also similar to the human protein, so that we could generate CAR-expressing T cells using our human-specific construct. The primary aim of the FIDO trial was to generate safety data for a planned FTIM study, as a previous trial with HER2 CAR-T cells reported a lethal incident [53]. All adverse events were mild and exclusively associated with IL-2 treatment. Toxicity was concentrated in the GI tract, with loose stool, decreased appetite, and vomiting. The eosinophilia observed has been described previously and is likely due to the release of IL-5 and GM-CSF release by IL-2-stimulated CD4-positive lymphocytes [54]. Reduced dosing of IL-2 resulted in control of toxicity, which was completely resolved after the termination of IL-2 administration.

In the safety FIDO trial, we also observed some signs of anti-tumoral activity, as evidenced by a near partial response in a dog with melanoma and potentially a delay in recurrence in a second dog. We are cautiously optimistic about these early signs of efficacy, although mindful of the pitfalls and caveats of the experiments. First, since our mouse data comparing the efficacy of TILs and CAR-T cells in NOG vs. hIL2-NOG mice undeniably indicated that IL-2 is essential for the efficacy of these therapies, we treated the dogs with subcutaneous injections of IL-2. It is therefore not inconceivable that some of the therapeutic effects observed were mediated by this factor, or that the gastrointestinal adverse events resulted in metabolic effects on the tumor. Second, we could not detect CAR-TILs in the blood of dogs using PCR. This could be due to the CAR-TILs not surviving or expanding, but it could also be due to them leaving the bloodstream because they express homing receptors for inflamed areas, such as tumors [41,42]. A future study protocol will need to include metastasectomy or biopsy after CAR-TIL therapy to evaluate whether CAR-TILs reach the target, as they commonly do in humans.

## 5. Conclusions

We present a novel approach to treating metastatic cancer in mice and dogs by combining CAR-T cell therapy and adoptive T cell transfer, called CAR-TIL treatment. CAR-TILs elicited complete eradication of tumors in immune-humanized PDX mouse models of melanoma, even in the absence of antigen presentation, indicating the potential usefulness of this strategy for treating melanomas that do not respond to existing therapies. This regimen should be tested in patients with metastatic melanoma to assess the potential of this therapy. Since owners and veterinarians of companion dogs with melanoma have difficulty accessing effective immunotherapies when they progress on approved therapies, the data here provide an interesting avenue for future trial activities. Companion dogs also contribute to 3R by reducing (and long-term replacing) the use of healthy beagles for safety studies and refining, since companion dogs have spontaneous tumor formation and are therefore more representative of patients in FTIM studies than using beagles.

## Figures and Tables

**Table 1:** Clinical responses to CAR-TIL treatment of Dog 3. Abbreviations used: CRP, C-reactive protein, and WBC, white blood cell count.

Days after Treatment	Treatment	Number of Cells/kg	CAR Expression	Tumor Size (mm)	CRP (mg/L)	WBC (109/L)	Eosinophils (109/L)	Side Effects
0	Dose 1: CAR-TILs	0.1 million	70% (mRNA)		<7	5	0.3	
14	Dose 2: CAR-TILs	10 million	75% (mRNA)	15 × 13	<7	8.4	0.4	Diarrhea that resolved after one day
35				27 × 14	<7	7.9	0.5	
63				35 × 29	<7	7.4	0.4	
78	Dose 3: CAR-TILs	10 million	1–7% (virus)	42 × 24	<7	7.2	0.6	
78	IL-2 injections 30,000 IU/kg q12h							Diarrhea and vomiting, decreased appetite all grade 1
84	IL-2 injections 30,000 IU/kg q12h			36 × 22	58	10.5	1.3	Eosinophilia grade 1
89	IL-2 injections 30,000 IU/kg q12h			34 × 24	51	23.2	9.7	Eosinophilia grade 1
112				52 × 44	<7	11.2		
133	Euthanized due to tumor progression							

**Table 2:** Clinical responses to CAR-TIL treatment of Dog 4.

Days after Treatment	Treatment	Number of Cells/kg	CAR Expression	CRP (mg/L)	WBC (109/L)	Eosinophils (109/L)	Side Effects
0	Dose 1: CAR-TILs	0.1 million	79% (mRNA)	<7	6.6	0.5	
20	Dose 2: CAR-TILs	10 million	9% (virus)	<7	4.4	0.3	
21–24	IL-2 injections 30,000 IU/kg q12h						Altered appetite, vomiting and diarreha all grade 1
27–32	IL-2 injections 30,000 IU/kg q24h			43	12.9	2.5	Eosinophilia, altered appetite, vomiting, diarreha and lethargy all grade 1
34				12	22.1	10.9	Eosinophilia grade 1
241							Revisit, in CR
290							Revisit, still in CR

## Data Availability

All sequencing data was deposited to the European Genome Archives and made available under controlled data access.

## Supplementary Materials

The following supporting information can be downloaded at: https://www.mdpi.com/article/10.3390/cancers15030648/s1, Figure S1: Differential expression of chemokine and adhesion between TILs and blood-derived T cells.; Figure S2: anti-HER2 CAR-expression after mRNA electroporation; Figure S3: Cytotoxicity of CAR-TILs and TILs on autologous tumor cells; Figure S4: Flow analysis of B2M in KO cells; Figure S5: Expression of anti-HER2 CAR in virus transduced melanoma TILs; Figure S6: CAR-TIL activity against melanoma xenografts in IL2 transgenic NOG mice; Tables S1 and S2: Clinical responses to CAR-TIL treatment of Dog 1 and 2.

## References

[1] Balch C.M., Gershenwald J.E., Soong S.-J., Thompson J.F., Atkins M.B., Byrd D.R., Buzaid A.C., Cochran A.J., Coit D.G., Ding S. (2009). Final Version of 2009 AJCC Melanoma Staging and Classification. J. Clin. Oncol..

[2] Hauschild A., Grob J.J., Demidov L.V., Jouary T., Gutzmer R., Millward M., Rutkowski P., Blank C.U., Miller Jr. W. (2012). H.; Kaempgen, E.; et al. Dabrafenib in BRAF-mutated metastatic melanoma: A multicentre, open-label, phase 3 randomised controlled trial. Lancet.

[3] Sosman J.A., Kim K.B., Schuchter L., Gonzalez R., Pavlick A.C., Weber J.S., McArthur G.A., Hutson T.E., Moschos S.J., Flaherty K.T. (2012). Survival in BRAF V600-mutant advanced melanoma treated with vemurafenib. N. Engl. J. Med..

[4] Flaherty K.T., Infante J.R., Daud A., Gonzalez R., Kefford R.F., Sosman J., Hamid O., Schuchter L., Cebon J., Ibrahim N. (2012). Combined BRAF and MEK inhibition in melanoma with BRAF V600 mutations. N. Engl. J. Med..

[5] Long G.V., Stroyakovskiy D., Gogas H., Levchenko E., de Braud F., Larkin J., Garbe C., Jouary T., Hauschild A., Grob J.J. (2014). Combined BRAF and MEK inhibition versus BRAF inhibition alone in melanoma. N. Engl. J. Med..

[6] Hodi F.S., O’Day S.J., McDermott D.F., Weber R.W., Sosman J.A., Haanen J.B., Gonzalez R., Robert C., Schadendorf D., Hassel J.C. (2010). Improved Survival with Ipilimumab in Patients with Metastatic Melanoma. N. Engl. J. Med..

[7] Robert C., Thomas L., Bondarenko I., O’Day S., Weber J., Garbe C., Lebbe C., Baurain J.-F., Testori A., Grob J.-J. (2011). Ipilimumab plus Dacarbazine for Previously Untreated Metastatic Melanoma. N. Engl. J. Med..

[8] Robert C., Long G.v., Brady B., Dutriaux C., Maio M., Mortier L., Hassel J.C., Rutkowski P., McNeil C., Kalinka-Warzocha E. (2015). Nivolumab in previously untreated melanoma without BRAF mutation. N. Engl. J. Med..

[9] Tawbi H.A., Schadendorf D., Lipson E.J., Ascierto P.A., Matamala L., Gutiérrez E.C., Rutkowski P., Gogas H.J., Lao C.D., De Menezes J.J. (2022). Relatlimab and Nivolumab versus Nivolumab in Untreated Advanced Melanoma. N. Engl. J. Med..

[10] Andersen R., Donia M., Ellebaek E., Borch T.H., Kongsted P., Iversen T.Z., Hölmich L.R., Hendel H.W., Met O., Andersen M.H. (2016). Long-Lasting Complete Responses in Patients with Metastatic Melanoma after Adoptive Cell Therapy with Tumor-Infiltrating Lymphocytes and an Attenuated IL2 Regimen. Clin. Cancer Res..

[11] Rosenberg S.A., Yang J.C., Sherry R.M., Kammula U.S., Hughes M.S., Phan G.Q., Citrin D.E., Restifo N.P., Robbins P.F., Wunderlich J.R. (2011). Durable complete responses in heavily pretreated patients with metastatic melanoma using T-cell transfer immunotherapy. Clin. Cancer Res..

[12] Rosenberg S.A., Packard B.S., Aebersold P.M., Solomon D., Topalian S.L., Toy S.T., Simon P., Lotze M.T., Yang J.C., Seipp C.A. (1988). Use of Tumor-Infiltrating Lymphocytes and Interleukin-2 in the Immunotherapy of Patients with Metastatic Melanoma. N. Engl. J. Med..

[13] Chang A.E., Karnell L.H., Menck H.R. (1998). The national cancer data base report on cutaneous and noncutaneous melanoma: A summary of 84,836 cases from the past decade. Cancer.

[14] Seibel I., Cordini D., Rehak M., Hager A., Riechardt A.I., Böker A., Heufelder J., Weber A., Gollrad J., Besserer A. (2015). Local Recurrence After Primary Proton Beam Therapy in Uveal Melanoma: Risk Factors, Retreatment Approaches, and Outcome. Am. J. Ophthalmol..

[15] Kujala E., Mäkitie T., Kivelä T. (2003). Very Long-Term Prognosis of Patients with Malignant Uveal Melanoma. Investig. Ophthalmol. Vis. Sci..

[16] Diener-West M., Reynolds S.M., Agugliaro D.J., Caldwell R., Cumming K., Earle J.D., Hawkins B.S., Hayman J.A., Jaiyesimi I., Jampol L.M. (2005). Development of metastatic disease after enrollment in the COMS trials for treatment of choroidal melanoma: Collaborative Ocular Melanoma Study Group Report No. 26. Arch. Ophthalmol..

[17] Leyvraz S., Konietschke F., Peuker C., Schütte M., Kessler T., Ochsenreither S., Ditzhaus M., Sprünken E.D., Dörpholz G., Lamping M. (2022). Biomarker-driven therapies for metastatic uveal melanoma: A prospective precision oncology feasibility study. Eur. J. Cancer.

[18] Piulats J.M., Espinosa E., de la Cruz M.L., Varela M., Alonso C.L., Martin-Algarra S., Lopez Castro R., Curiel T., Rodriguez-Abreu D., Redrado M. (2021). Nivolumab Plus Ipilimumab for Treatment-Naive Metastatic Uveal Melanoma: An Open-Label, Multicenter, Phase II Trial by the Spanish Multidisciplinary Melanoma Group (GEM-1402). J. Clin. Oncol..

[19] Pelster M.S., Gruschkus S.K., Bassett R., Gombos D.S., Shephard M., Posada L., Glover M.S., Simien R., Diab A., Hwu P. (2021). Nivolumab and Ipilimumab in Metastatic Uveal Melanoma: Results from a Single-Arm Phase II Study. J. Clin. Oncol..

[20] Wolchok J.D., Chiarion-Sileni V., Gonzalez R., Rutkowski P., Grob J.-J., Cowey C.L., Lao C.D., Wagstaff J., Schadendorf D., Ferrucci P.F. (2017). Overall Survival with Combined Nivolumab and Ipilimumab in Advanced Melanoma. N. Engl. J. Med..

[21] Khoja L., Atenafu E., Suciu S., Leyvraz S., Sato T., Marshall E., Keilholz U., Zimmer L., Patel S., Piperno-Neumann S. (2019). Meta-analysis in metastatic uveal melanoma to determine progression free and overall survival benchmarks: An international rare cancers initiative (IRCI) ocular melanoma study. Ann. Oncol..

[22] Olofsson B.R., Nelson A., Shafazand A., All-Ericsson C., Cahlin C., Elander N., Helgadottir H., Kiilgaard J.F., Kinhult S., Ljuslinder I. (2022). Isolated hepatic perfusion as a treatment for uveal melanoma liver metastases, first results from a phase III randomized controlled multicenter trial (the SCANDIUM trial). J. Clin. Oncol..

[23] Zager J.S., Orloff M.M., Ferrucci P.F., Glazer E.S., Ejaz A., Richtig E., Ochsenreither S., Lowe M.C., Reddy S.A., Beasley G. (2022). FOCUS phase 3 trial results: Percutaneous hepatic perfusion (PHP) with melphalan for patients with ocular melanoma liver metastases (PHP-OCM-301/301A). J. Clin. Oncol..

[24] Ny L., Jespersen H., Karlsson J., Alsén S., Filges S., All-Eriksson C., Andersson B., Carneiro A., Helgadottir H., Levin M. (2021). The PEMDAC phase 2 study of pembrolizumab and entinostat in patients with metastatic uveal melanoma. Nat. Commun..

[25] Chandran S.S., Somerville R.P.T., Yang J.C., Sherry R.M., Klebanoff C.A., Goff S.L., Wunderlich J.R., Danforth D.N., Zlott D., Paria B.C. (2017). Treatment of metastatic uveal melanoma with adoptive transfer of tumour-infiltrating lymphocytes: A single-centre, two-stage, single-arm, phase 2 study. Lancet Oncol..

[26] Middleton M.R., McAlpine C., Woodcock V.K., Corrie P., Infante J.R., Steven N.M., Evans T.R.J., Anthoney A., Shoushtari A.N., Hamid O. (2020). Tebentafusp, a TCR/anti-CD3 bispecific fusion protein targeting gp100, potently activated anti-tumor immune responses in patients with metastatic melanoma. Clin. Cancer Res..

[27] Nathan P., Hassel J.C., Rutkowski P., Baurain J.-F., Butler M.O., Schlaak M., Sullivan R.J., Ochsenreither S., Dummer R., Kirkwood J.M. (2021). Overall Survival Benefit with Tebentafusp in Metastatic Uveal Melanoma. N. Engl. J. Med..

[28] Olivier T., Prasad V. (2022). Tebentafusp in first-line melanoma trials: An outperforming outlier. Transl. Oncol..

[29] Slingluff C.L., Colella T.A., Thompson L., Graham D.D., Skipper J.C., Caldwell J., Brinckerhoff L., Kittlesen D.J., Deacon D.H., Oei C. (2000). Melanomas with concordant loss of multiple melanocytic differentiation proteins: Immune escape that may be overcome by targeting unique or undefined antigens. Cancer Immunol. Immunother..

[30] Drake C.G., Jaffee E., Pardoll D.M. (2006). Mechanisms of Immune Evasion by Tumors. Adv Immunol..

[31] Maude S.L., Laetsch T.W., Buechner J., Rives S., Boyer M., Bittencourt H., Bader P., Verneris M.R., Stefanski H.E., Myers G.D. (2018). Tisagenlecleucel in Children and Young Adults with B-Cell Lymphoblastic Leukemia. N. Engl. J. Med..

[32] Majzner R.G., Mackall C.L. (2019). Clinical lessons learned from the first leg of the CAR T cell journey. Nat. Med..

[33] Aparicio S., Hidalgo M., Kung A. (2015). Examining the utility of patient-derived xenograft mouse models. Nat. Rev. Cancer.

[34] Paoloni M., Khanna C. (2008). Translation of new cancer treatments from pet dogs to humans. Nat. Rev. Cancer.

[35] Bonnett B.N., Egenvall A., Hedhammar A., Olson P. (2005). Mortality in over 350,000 insured Swedish dogs from 1995-2000: I. Breed-, gender-, age- and cause-specific rates. Acta Veter Scand..

[36] Gordon I., Paoloni M., Mazcko C., Khanna C. (2009). The Comparative Oncology Trials Consortium: Using Spontaneously Occurring Cancers in Dogs to Inform the Cancer Drug Development Pathway. PLOS Med..

[37] Pazzi P., Steenkamp G., Rixon A.J. (2022). Treatment of Canine Oral Melanomas: A Critical Review of the Literature. Vet. Sci..

[38] Wong K., van der Weyden L., Schott C.R., Foote A., Constantino-Casas F., Smith S., Dobson J.M., Murchison E.P., Wu H., Yeh I. (2019). Cross-species genomic landscape comparison of human mucosal melanoma with canine oral and equine melanoma. Nat. Commun..

[39] Prouteau A. (2019). Canine Melanomas as Models for Human Melanomas: Clinical, Histological, and Genetic Comparison. Genes.

[40] Forsberg E., Lindberg M.F., Jespersen H., Alsén S., Bagge R.O., Donia M., Svane I.M., Nilsson O., Ny L., Nilsson L.M. (2019). HER2 CAR-T Cells Eradicate Uveal Melanoma and T-cell Therapy-Resistant Human Melanoma in IL2 Transgenic NOD/SCID IL2 Receptor Knockout Mice. Cancer Res..

[41] Dudley M.E., Wunderlich J.R., Robbins P.F., Yang J.C., Hwu P., Schwartzentruber D.J., Topalian S.L., Sherry R., Restifo N.P., Hubicki A.M. (2002). Cancer regression and autoimmunity in patients after clonal repopulation with antitumor lymphocytes. Science.

[42] Jespersen H., Lindberg M.F., Donia M., Söderberg E.M.V., Andersen R., Keller U., Ny L., Svane I.M., Nilsson L.M., Nilsson J.A. (2017). Clinical responses to adoptive T-cell transfer can be modeled in an autologous immune-humanized mouse model. Nat. Commun..

[43] Einarsdottir B.O., Bagge R.O., Bhadury J., Jespersen H., Mattsson J., Nilsson L.M., Truvé K., López M.D., Naredi P., Nilsson O. (2014). Melanoma patient-derived xenografts accurately model the disease and develop fast enough to guide treatment decisions. Oncotarget.

[44] Karlsson J., Nilsson L.M., Mitra S., Alsén S., Shelke G.V., Sah V.R., Forsberg E.M.V., Stierner U., All-Eriksson C., Einarsdottir B. (2020). Molecular profiling of driver events in metastatic uveal melanoma. Nat. Commun..

[45] Nguyen S.M., Thamm D., Vail D.M., London C.A. (2013). Response evaluation criteria for solid tumours in dogs (v1.0): A Veterinary Cooperative Oncology Group (VCOG) consensus document. Vet. Comp. Oncol..

[46] LeBlanc A.K., Atherton M., Bentley R.T., Boudreau C.E., Burton J.H., Curran K.M., Dow S., Giuffrida M.A., Kellihan H.B., Mason N.J. (2021). Veterinary Cooperative Oncology Group—Common Terminology Criteria for Adverse Events (VCOG-CTCAE v2) following investigational therapy in dogs and cats. Vet. Comp. Oncol..

[47] Butler A., Hoffman P., Smibert P., Papalexi E., Satija R. (2018). Integrating single-cell transcriptomic data across different conditions, technologies, and species. Nat. Biotechnol..

[48] Dobin A., Davis C.A., Schlesinger F., Drenkow J., Zaleski C., Jha S., Batut P., Chaisson M., Gingeras T.R. (2013). STAR: Ultrafast universal RNA-seq aligner. Bioinformatics.

[49] Anders S., Pyl P.T., Huber W. (2015). HTSeq—A Python framework to work with high-throughput sequencing data. Bioinformatics.

[50] Bagge R.O., Demir A., Karlsson J., Alaei-Mahabadi B., Einarsdottir B.O., Jespersen H., Lindberg M.F., Muth A., Nilsson L.M., Persson M. (2018). Mutational Signature and Transcriptomic Classification Analyses as the Decisive Diagnostic Tools for a Cancer of Unknown Primary. JCO Precis. Oncol..

[51] Mills J.K., Henderson M.A., Giuffrida L., Petrone P., Westwood J.A., Darcy P.K., Neeson P.J., Kershaw M.H., Gyorki D.E. (2021). Generating CAR T cells from tumor-infiltrating lymphocytes. Ther. Adv. Vaccines Immunother..

[52] Sharpless N.E., DePinho R. (2006). The mighty mouse: Genetically engineered mouse models in cancer drug development. Nat. Rev. Drug Discov..

[53] Morgan R.A., Yang J.C., Kitano M., E Dudley M., Laurencot C.M. (2010). A Rosenberg. Case Report of a Serious Adverse Event Following the Administration of T Cells Transduced with a Chimeric Antigen Receptor Recognizing ERBB2. Mol. Ther..

[54] Macdonald D., Gordon A.A., Kajitani H., Enokihara H., Barrett A.J. (1990). Interleukin-2 treatment-associated eosinophilia is mediated by interleukin-5 production. Br. J. Haematol..

