# Supplementary material for: Treatment with Anti-HER2 Chimeric Antigen Receptor Tumor-Infiltrating Lymphocytes (CAR-TILs) Is Safe and Associated with Antitumor Efficacy in Mice and Companion Dogs
Source: Cancers (Basel). 2023 Jan 20;15(3):648. doi: 10.3390/cancers15030648 (PMC9913266; doi:10.3390/cancers15030648)
Supplement: Supplementary file 1 [file cancers-15-00648-s001.zip › cancers-2183100-supplementary.pdf]

Supplemental Figures and Tables

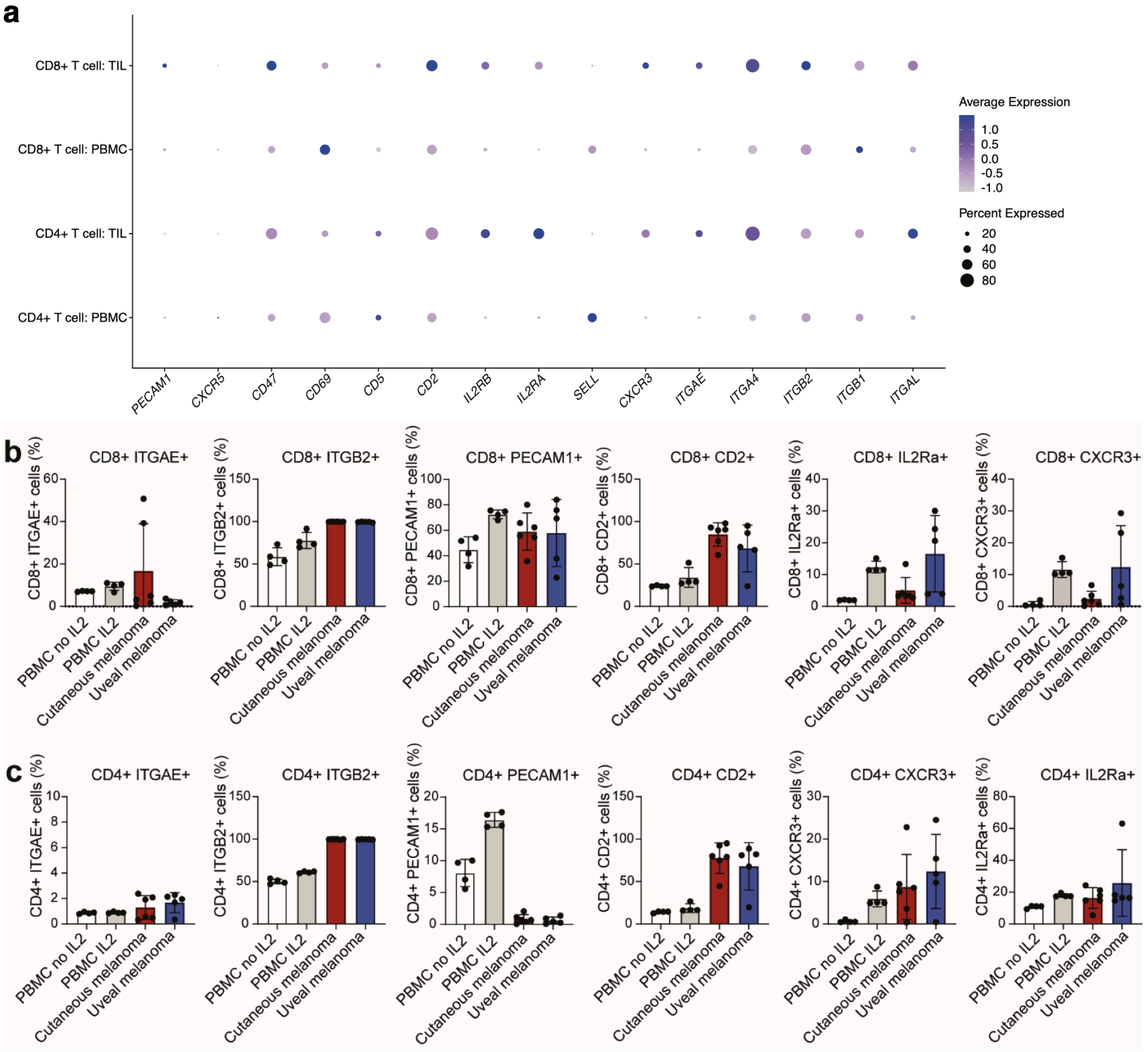

Supplemental Figure S1. Differential expression of chemokine and adhesion between TILs and blood-derived T cells.

(a) Differentially expressed genes at the single-cell level in the categories of chemokine receptors, ligands, or adhesion molecules between CD8^{+} and CD4^{+} TILs obtained from uveal melanoma patient samples and PBMC-derived T cells. Tests were carried out with the Seurat FindMarkers function, using logistic regression, adjusting for sex and cell cycle scores, as described in Methods. (b-c) Six of the differentially expressed genes were also analyzed by flow cytometry in both CD4^{+} (b) and CD8^{+} cells (c) from PBMC, skin melanoma or uveal melanoma.

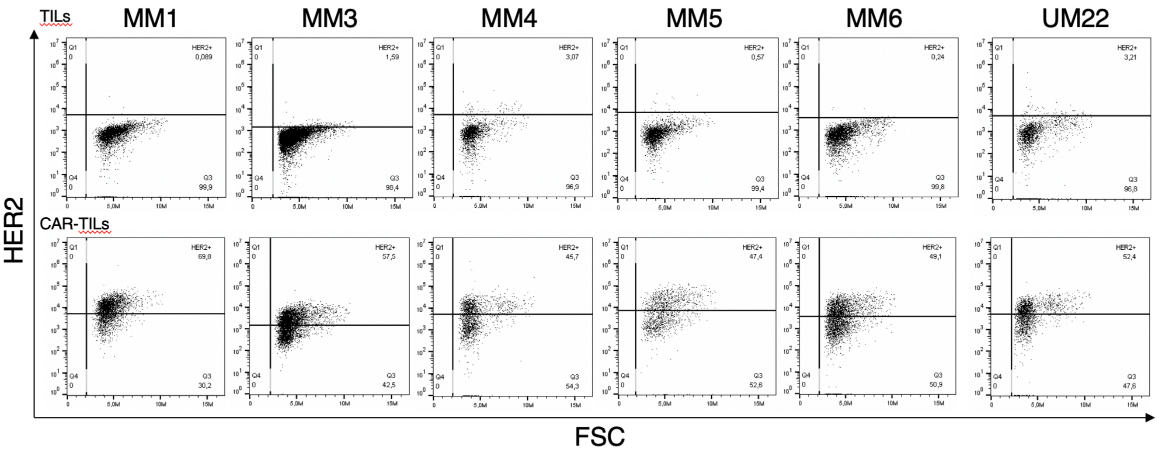

Supplemental Figure S2: anti-HER2 CAR-expression after electroporation

Flow cytometry data showing anti-HER2 CAR expression after anti-HER2 CAR mRNA electroporation by detecting bound biotinylated HER2 protein. Representative plots for samples MM1, MM3, MM4, MM5, MM6, and UM22.

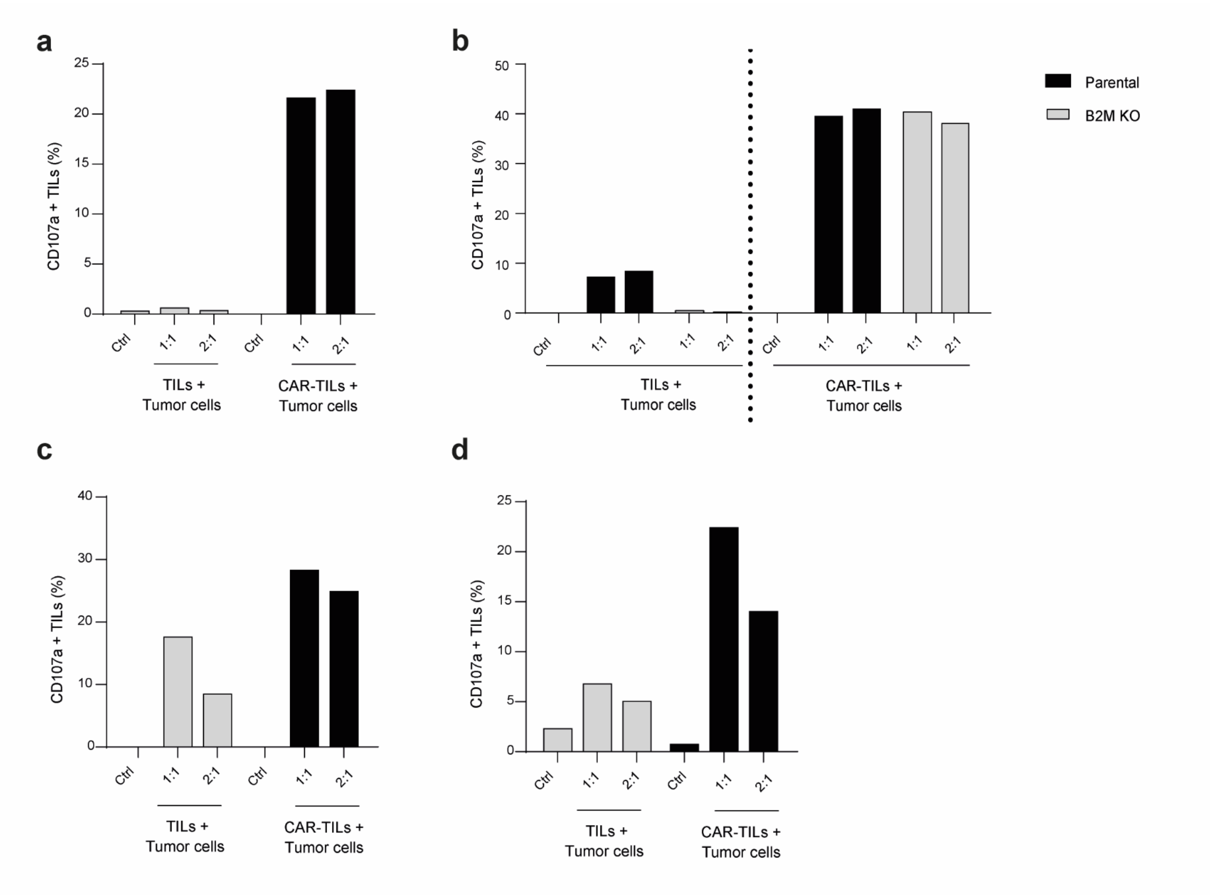

Supplemental Figure S3. CAR-TILs degranulate more potently than TILs on autologous tumor cells

Degranulation detected by CD107a staining in TILs and CAR-TILs after 4-6 hours co-culture with UM22 (a), MM3 (b), MM4 (c), and MM5 (d) autologous tumor cell lines. The analysis was performed in singlets twice, and representative data are shown from one of the experiments.

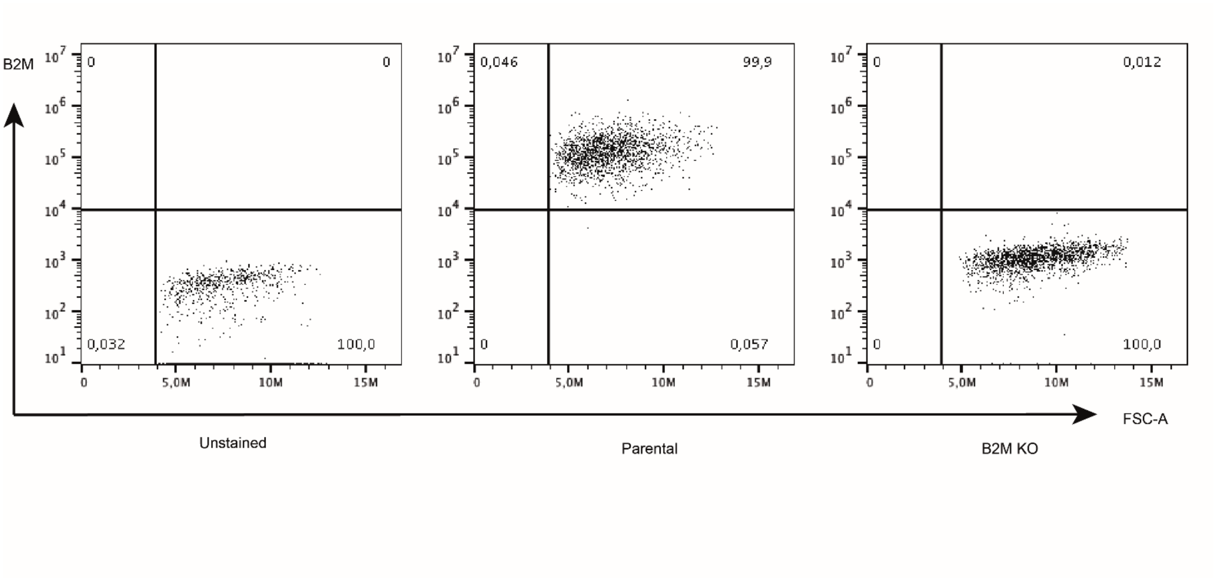

Supplemental Figure S4. B2M KO analysis

CRISPR/Cas9 technology has been used for genetic disruption of *B2M*. Cells were stained with a B2M-PE antibody, separated by magnetic sorting based on negative binding to PE-beads, and subsequently further selected by killing off B2M positive cells *in vitro* for 48 h with autologous TILs. The resulting B2M negative cell population (*B2M* KO) and parental cells (wt) were stained with a B2M antibody and analyzed using flow cytometry.

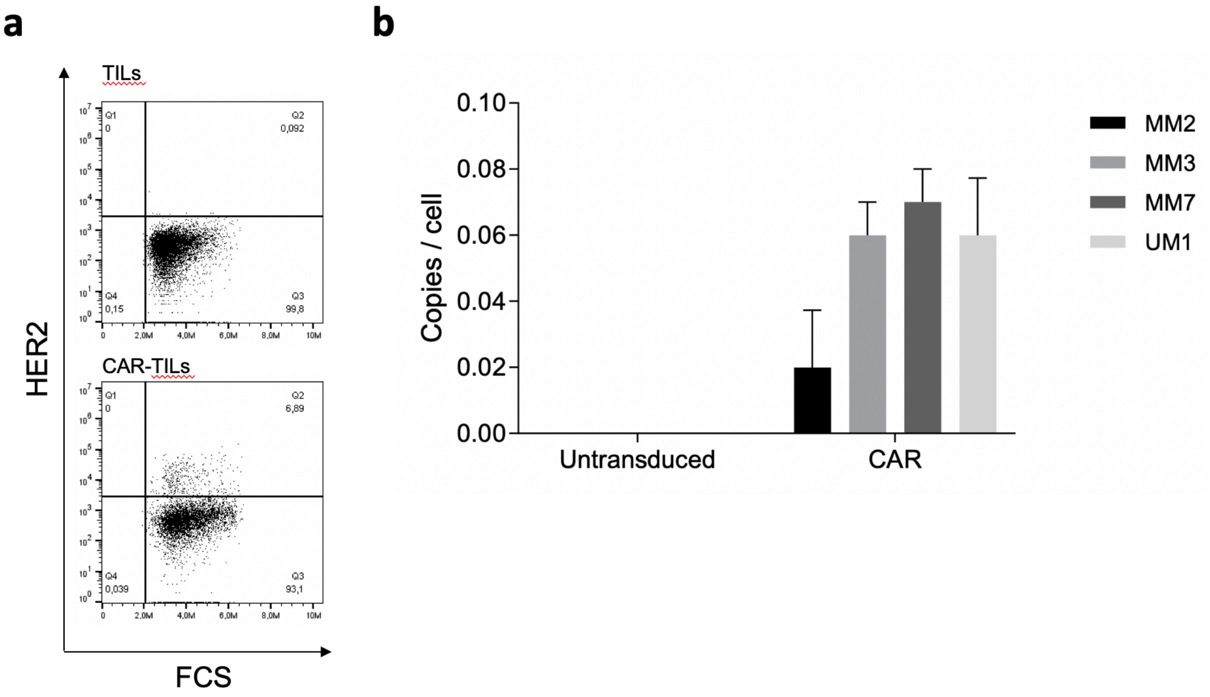

Supplemental Figure S5. Expression of anti-HER2 CAR in melanoma TILs

(a) CAR expression in MM3 TILs (untransduced) and CAR-TILs (CAR) detected by flow cytometry. (b) qPCR in samples MM2, MM3, MM7, and UM1. The data are presented as the mean ± SD of three replicates.

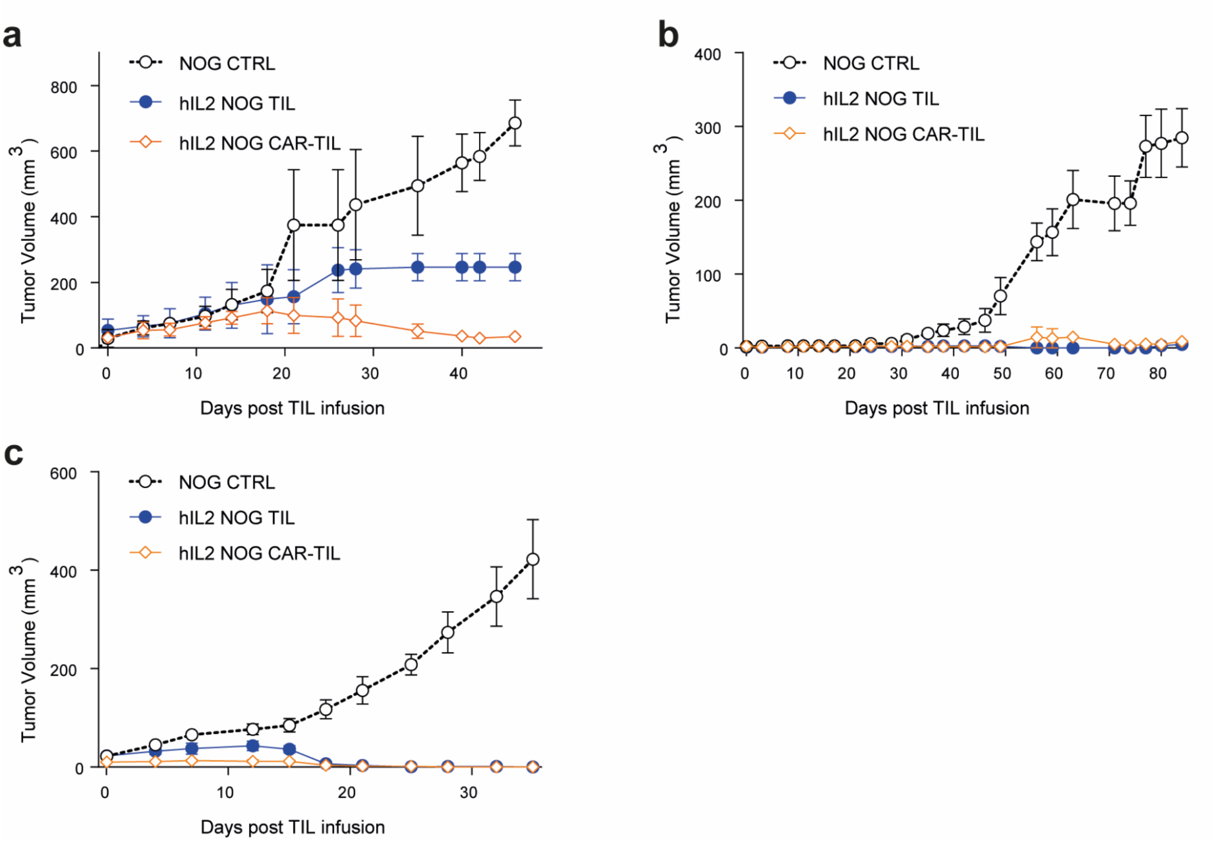

Supplemental Figure S6. CAR-TILs can be used to treat autologous melanoma xenografts in IL2 transgenic NOG mice

TILs and lentivirally transduced CAR-TILs were produced from three melanoma samples and used to treat tumour-bearing mice. Two samples were from cutaneous melanoma MM2 (a) and MM7 (c) and one was from uveal melanoma UM1 (b).

Supplemental Tables

Supplemental Table S1. Clinical responses to CAR-TIL treatment of Dog 1

Days from treatment	Treatment	Number of cells/kg	CAR expression	Tumor size (mm)	CRP (mg/l)	WBC (10^9/l)	Side effects
0	Dose 1: CAR-TILs	0.1 million	0.5-1.6% (virus)	60 x 50	<7	5.2	
8				45 x 48	<7	5.8	
28	Dose 2: CAR-TILs	1 million	0.4% (virus)	40 x 40	<7	10.5	
50					265	2.7	
51	Euthanized due to complications to an acute perforated pyometra						(none reported)

Supplemental Table S2. Clinical responses to CAR-TIL treatment of Dog 2

Days from treatment	Treatment	Number of cells/kg	CAR expression	Tumor size (mm)	CRP (mg/l)	WBC (10^9/l)	Side effects
0	Dose 1: CAR-TILs	0.1 million	33% (mRNA)	NA			
7					9	11.2	
19	Dose 2: CAR-TILs	5 million	29% (mRNA)	NA	7	9.6	
28					21	12.3	
42	Euthanized due to progressive tumor disease			NA	41	20.5	(none reported)

Dataset (Excel file)

Supplemental Table S3. Difference in expression of homing receptors in blood-derived and tumor derived T-cells

Statistical details for the differential expression analysis of single-cell RNA sequencing data are shown **Fig. S1a**.
